# Comparing and Contrasting the Multiple Roles of Butenolide Plant Growth Regulators: Strigolactones and Karrikins in Plant Development and Adaptation to Abiotic Stresses

**DOI:** 10.3390/ijms20246270

**Published:** 2019-12-12

**Authors:** Tao Yang, Yuke Lian, Chongying Wang

**Affiliations:** Ministry of Education Key Laboratory of Cell Activities and Stress Adaptations, School of Life Sciences, Lanzhou University, Lanzhou 730000, China; yangtao@lzu.edu.cn (T.Y.); lianyk18@lzu.edu.cn (Y.L.)

**Keywords:** strigolactone, karrikin, development, abiotic stress, hormonal crosstalk

## Abstract

Strigolactones (SLs) and karrikins (KARs) are both butenolide molecules that play essential roles in plant growth and development. SLs are phytohormones, with SLs having known functions within the plant they are produced in, while KARs are found in smoke emitted from burning plant matter and affect seeds and seedlings in areas of wildfire. It has been suggested that SL and KAR signaling may share similar mechanisms. The α/β hydrolases DWARF14 (D14) and KARRIKIN INSENSITIVE 2 (KAI2), which act as receptors of SL and KAR, respectively, both interact with the F-box protein MORE AXILLARY GROWTH 2 (MAX2) in order to target SUPPRESSOR OF MAX2 1 (SMAX1)-LIKE/D53 family members for degradation via the 26S proteasome. Recent reports suggest that SLs and/or KARs are also involved in regulating plant responses and adaptation to various abiotic stresses, particularly nutrient deficiency, drought, salinity, and chilling. There is also crosstalk with other hormone signaling pathways, including auxin, gibberellic acid (GA), abscisic acid (ABA), cytokinin (CK), and ethylene (ET), under normal and abiotic stress conditions. This review briefly covers the biosynthetic and signaling pathways of SLs and KARs, compares their functions in plant growth and development, and reviews the effects of any crosstalk between SLs or KARs and other plant hormones at various stages of plant development. We also focus on the distinct responses, adaptations, and regulatory mechanisms related to SLs and/or KARs in response to various abiotic stresses. The review closes with discussion on ways to gain additional insights into the SL and KAR pathways and the crosstalk between these related phytohormones.

## 1. Introduction

Environmental constraints, both biotic and abiotic, can deliver deleterious effects to both plant survival and crop productivity [1,2,3]. Moreover, global climate change threatens greater environmental deterioration and risks the continued sustainability of agriculture [4,5]. Plants have evolved many survival strategies to respond to various adverse environmental conditions, including morphological changes, physiological, biochemical, and molecular responses, including global reprogramming of transcription [2,6,7,8,9,10,11]. Various phytohormones, such as abscisic acid (ABA), brassinosteroids (BRs), jasmonic acid (JA), salicylic acid (SA), ethylene, and cytokinins (CKs), integrate the signaling needed to cooperatively optimize both plant development and the adaptive responses to environmental stressors [12,13,14,15,16,17,18,19]. Plants are able to alleviate the adverse effects of biotic and abiotic environmental factors through interactions between the phytohormone regulatory networks via the perception and signal transduction originating at various receptors [20,21,22,23].

Strigolactones (SLs) were originally isolated from root exudates of cotton and as seed germination stimulants from plants in the Orobanchaceae family that parasitize plant roots (*Striga*, *Phelipanche*, and *Orobanche spp.*) [24,25,26]. SLs normally control seed germination and seedling development [27], shoot branching [28,29,30,31,32], root architecture [33], and leaf senescence [34]. SLs also promote beneficial symbiotic relationships between host plants and mycorrhizal fungi [35,36]. The biosynthesis and signaling of SLs are regulated by various abiotic stress factors [37,38,39,40], including the recently reported SL involvement in responding to nutrient deprivation, drought, chilling and salinity [38,40,41,42,43,44,45,46,47,48,49,50]. Such studies provide new insights into the novel roles SL signaling plays in the regulation of plant adaptation to adverse environmental conditions [51,52,53,54].

Karrikins (KARs) are found in smoke released from the heating or combustion of plant material, after which they can stimulate the germination of dormant seeds [55,56,57,58]. KARs are also involved in the inhibition of hypocotyl elongation and in the promotion of cotyledon expansion and seedling vigor [59,60,61]. KARs are structurally related to SLs and share a common substituted butenolide moiety [55,59,62]. Recent studies also provided evidence that KARs have potential functions in mediating abiotic stress tolerance in plants [8,63,64]. These finding suggest that KAR molecules may have a similar function to those of SLs in plant adaptation to abiotic stress.

Previous studies and reviews have summarized the biosynthesis, perception and signaling of SLs and KARs [52,53,65,66]. In addition, the functions of SL and KAR signaling in the control of plant growth and development have also been described [62]. Numerous studies have particularly focused on the functioning of SLs and KARs in plant responses and adaptation to abiotic stress. Despite a basic structural similarity, KARs and SLs are not interchangeable signals. In this review, we briefly cover their biosynthesis and signaling networks, compare their functions in plant growth and development, and highlight the putative mechanisms by which SLs and/or KARs regulate the response to abiotic stresses. Furthermore, the crosstalk between SLs, KARs and other phytohormones under adverse conditions are also discussed.

## 2. Classical Structure and Biosynthesis of SLs and KARs

**Figure 1:**
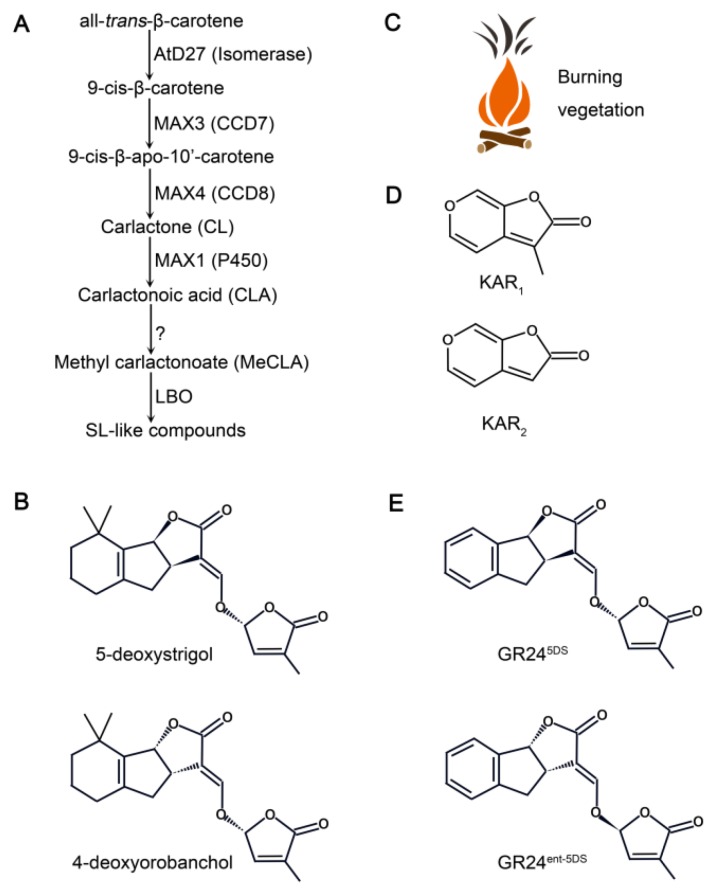
Strigolactone (SL) biosynthesis in Arabidopsis and chemical structures of SLs and karrikins (KARs). (**A**) Proposed model for SL biosynthesis in Arabidopsis. The conversion of carlactone (CL) from all-*trans*-β-carotene by the sequential actions of the isomerase AtD27 and the carotenoid cleavage dioxygenases MAX3 and MAX4 in plastids. In the cytosol, CL is converted into SLs via the cytosolic P450 MAX1, LATERAL BRANCHING OXIDOREDUCTASE (LBO) and other unknown enzymes. (**B**) Structures of two representatives of natural SLs (5-deoxystrigol and 4-deoxyrobanchol). (**C**) KARs are produced during the burning of vegetation. (**D**) Structures of the two major KARs (KAR_1_ and KAR_2_). (**E**) Structures of the commonly used synthetic SL analog *rac*-GR24, which is a mixture of GR24^5DS^ and its enantiomer GR24*^ent-^*^5DS^.

SLs are carotenoid-derived phytohormones (Figure 1). Carotenoids are converted by the sequential action of all-*trans*-β-carotene isomerase (AtD27 in Arabidopsis and D27 in rice) [67,68,69], two carotenoid cleavage dioxygenases: [CCD7 (MAX3 in Arabidopsis, RMS5 in Pea, DAD3 in Petunia and HTD1/D17 in rice)] [70,71,72], and CCD8 (MAX4 in Arabidopsis, RMS1 in Pea, DAD1 in Petunia and D10 in rice) [73,74,75,76] into the SL intermediate carlactone (CL) [77]. CL is oxidized by the cytochrome P450 enzyme MAX1 [Carlactone oxidase (Os01g0700900) and orobanchol synthase (Os01g0701400) in rice] [78,79,80] and subsequently methylated and oxidized by lateral branching oxidoreductase (LBO), which may catalyze the final step in SL-like compound biosynthesis in Arabidopsis [81] (Figure 1).

KARs are small organic chemicals with bioactive compounds identified as butenolides, which are related to chemical 3-methyl-2H-furo[2,3-c]pyran-2-one [55,57,58]. KARs are produced by the pyrolysis of simple carbohydrates, such as xylose, glucose, or cellulose, which can occur during wildfires [82] (Figure 1). To date, six KAR compounds, annotated as KAR_1_ to KAR_6_, have been identified in plant-derived smoke and differ in their methyl group substitutions [57]. Among them, KAR_2_ is commonly used in research due to its higher bioactivity in Arabidopsis compared to other KARs [83]. In general, KARs are stable at room temperature and in aqueous solutions [55,58].

## 3. Signal Transductions of SL and KAR

SLs and KARs belong to the butenolide class of compounds [55,67]. SLs and KARs have similar structures [84] and play roles in the growth and development of plants. Recent, significant breakthroughs have been made that reveal the connection between SL and KAR signal transduction pathways.

**Figure 2:**
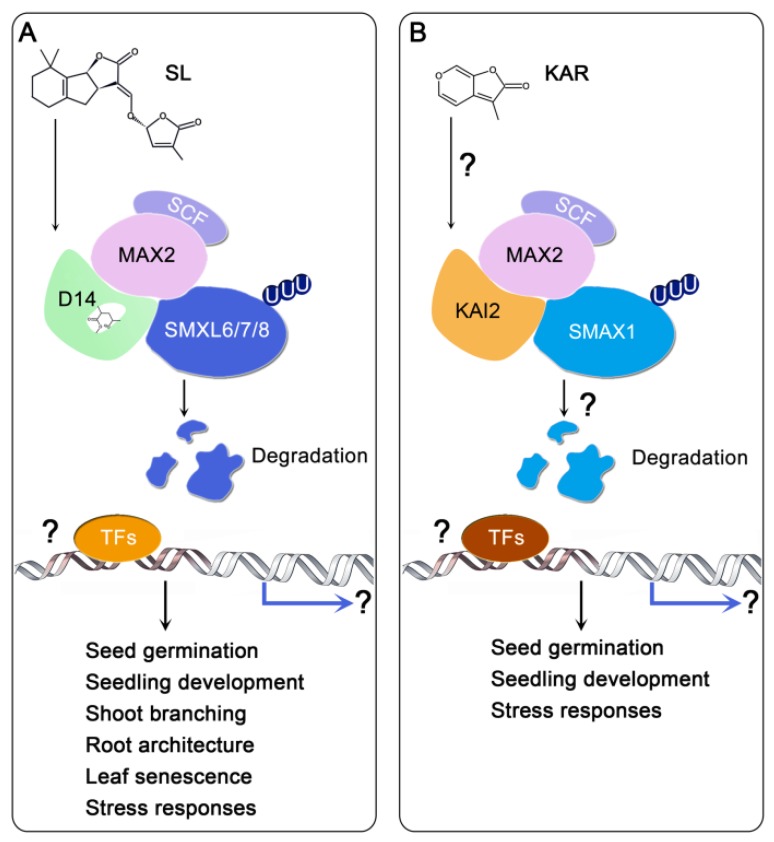
Simplified models of SL signaling and hypothetic KAR signaling. (**A**) The SL receptor AtD14 binds and hydrolyzes the SL, triggering the formation of a D14-SCF^MAX2^-SMXL6/7/8 complex which targets SMXL6/7/8 for ubiquitination and degradation, which then relieves the repression of yet-unknown TFs and activates the expression of downstream targets. (**B**) KAR or a putative KAI2 ligand is perceived through KAI2. The ligand–receptor interaction triggers the formation of a KAI2-SCF^MAX2^-SMXL1 complex to induce the ubiquitination and degradation of SMXL1, which then activates downstream responses. Question marks indicate the undemonstrated hypotheses. SL, strigolactone; KAR, karrikin; D14, DWARF14; MAX2, MORE AXILLARY GROWTH 2; SMAX1, SUPPRESSOR OF MAX2 1; SMXL, SMAX1-LIKE; KAI2, KARRIKIN INSENSITIVE 2; U, ubiquitin; TFs, transcription factors.

Recent crystallization studies have shown how SLs are perceived by D14 (DWARF14), a non-canonical α/β hydrolase receptor [85,86]. Upon binding, AtD14 docks SL into its catalytic pocket in an “open state” and hydrolyses SL into a hydrolytic D-ring-derived intermediate molecule (D3), which is then covalently and irreversibly sealed inside the closed catalytic cavity of AtD14 [86,87,88]. The conformation of AtD14-D3 is significantly different than that of AtD14 after the open-to-closed transition, and now interacts with the F-box leucine-rich repeat protein MAX2. The resulting MAX2/RMS4/D3 SCF complex targets the transcriptional repressors D53 (DWARF 53) in rice or SMXL6/7/8 (SUPPRESSOR OF MAX2 1 (SMAX1)-LIKE 6/7/8) in Arabidopsis for degradation via the 26S proteasome. Removal of D53/SMXL6/7/8 allows SL-based signal transduction, which alters various physiological and biochemical functions [30,32,86,89,90] (Figure 2A).

The α/β-fold hydrolase KARRIKIN INSENSITIVE2 (KAI2), which is a paralogue of D14, is characterized as the KAR receptor in Arabidopsis [91]. KAR_1_ has been demonstrated to bind KAI2 [91,92]. Although KAR signaling mechanisms remain unclear, genetic studies have provided evidence that the KAI2 signal pathway may be similar to that of D14 [84,93,94]. Based on an analogy to SL signaling, the hypothetical signaling for smoke-derived KARs or KAR-like (KL) metabolites initiates with its perception by KAI2, which leads to its interaction with MAX2 and SMXL1 and subsequent formation of a SCF^MAX2^-KAI2-SMXL1 complex. SMXL1, like SMXL6/7/8 in the SL pathway, is then polyubiquitinated for 26S proteasomal degradation, triggering the signaling downstream of the KARs [28,90]. However, this proposed KAI2-dependent signaling pathway, including the KAR-mediated interaction among KAI2, MAX2, and SMXL1 and the degradation of SMXL1, remain to be further confirmed (Figure 2B).

Since the signal transduction pathways following both KAR and SL are both dependent on MAX2, which interacts with both KAI2 and D14, crosstalk between KAR and SL signaling may be observed. In other words, MAX2 may act as a connector between the two different signaling pathways. However, the physiological responses are completely different, possibly because MAX2-dependent signaling can identify the different response signals. Thus, a comparative study of the KAR and SL pathways should be performed in the future to provide a better understanding of the functions of D14 and KAI2 in MAX2-dependent signaling. Although both D14 and KAI2 undergo degradation during signal transduction, there is evidence that the degradation of KAI2 might be dependent on its Ser95, and not on MAX2 or the 26S proteasome, implying that other unidentified components may contribute to the degradation, and possibly downstream signaling, of KAI2 [94,95].

## 4. Similarities and Differences Between the Functions of SLs and KARs in Plant Development

**Figure 3:**
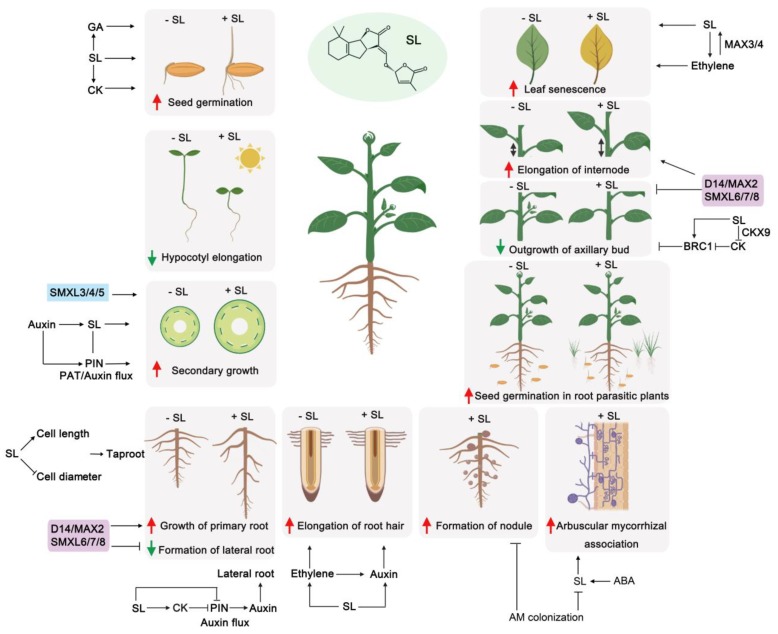
Roles of SLs in plant development. SLs interact with auxin, ABA, CK, GA and ethylene to regulate plant development at different stages. Red arrows represent a promotion effect or positive regulation, and green arrows represent inhibitory effects or negative regulation. GA, gibberellic acid; CK, cytokinins; ABA, abscisic acid. PIN1, PIN-FORMED 1; PAT, polar auxin transport; BRC1, BRANCH 1.

**Figure 4:**
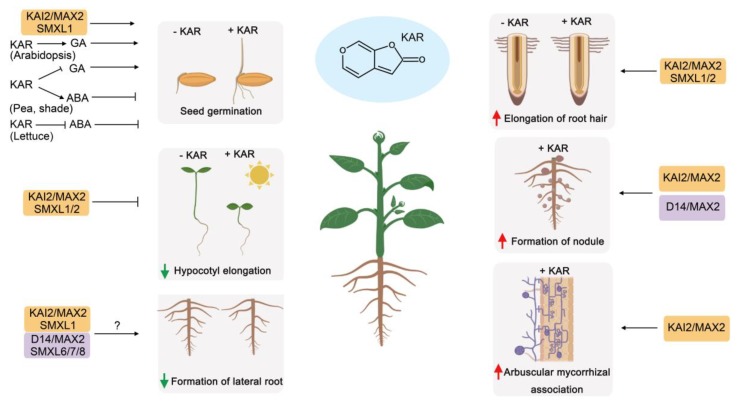
Roles of KARs in plant development. KARs interact with ABA, GA and other hormones to regulate plant development at different stages. Red arrows represent promotion effects/positive regulation, and green arrows represent inhibitory effect/negative regulation. GA, gibberellic acid; ABA, abscisic acid. Question marks (?) represent potential interactions that have not been directly demonstrated.

In recent years, numerous lines of evidence have demonstrated that SLs and KARs regulate plant growth and development. Although SLs and KARs have similar chemical structures, plants can still distinguish SL and KAR signaling at various stages of development. Some of the major effects on plant development initiated by SLs and KARs are highlighted below (Figure 3 and Figure 4).

### 4.1. Utilization of Storage Reserves in the Early Seed Germination

Seeds sense endogenous and environmental signals such as endogenous storage reserves, air, oxygen, temperature, water, light, or darkness to determine whether they are suitable for germination. These signals may play important roles in promoting or inhibiting germination. Chemical germination signals derived from smoke have attracted much attention due to their significant effects on the seed germination of many plants. Wildfire smoke contains certain potent bioactive compounds including KAR_1_, trimethylbutenolide (TMB) and smoke-water (SW). In particular, KAR may modulate the early seed germination by influencing the activity of hydrolase and contents of lipids, protein, carbohydrate, and starch during seed germination. In *Lactuca sativa* seeds, KAR_1_ and SW treatments enhance α-amylase activity in dark and FR light, however, TMB inhibits α-amylase activity in all light treatments. Similarly, KAR_1_ improves the contents of lipids, protein, carbohydrate, and starch, while TMB plays an opposite role in the early seed germination. These data suggest that the mobilization and utilization of storage reserves are improved after applying KAR_1_ and SW, and then, enough energy is provided for the germination of the seeds [96].

### 4.2. Seed Germination

Both SLs and KARs have been found to stimulate seed germination. The difference is that SLs are mainly secreted into the soil by the plant roots and end up stimulating the germination of parasitic plant seeds or transported upward through the xylem to the aboveground parts, while KARs are found in the smoke arising from fire and can then promote the germination of seeds on the ground after the fire [55,67]. SLs can alleviate the inhibition of seed germination by heat through regulating GA and ABA levels [97]. In addition to promoting the germination of parasitic plant seeds, SLs also inhibit *Physcomitrella* spore germination [98].

KARs can promote the light response during seed germination [83,99]. Surprisingly, KARs can delay soybean seed germination under shaded conditions, but not in the dark or under white light, by regulating the biosynthesis of ABA and GA [100]. A recent study in lettuce demonstrated that SW and KAR_1_ promote seed germination through decreasing the ABA content and enhancing the hydrolase activity, however, the KAR_1_-related compound TMB inhibits the germination of lettuce seeds by increasing ABA and inhibiting cytokinin contents under dark conditions [96].

### 4.3. Leaf Morphogenesis

Both SLs and KARs are involved in the regulation of leaf shape. Arabidopsis lines lacking SL signaling due to mutation of *max* or *d14* had a reduced petiole and leaf aspect ratio and smaller and rounder leaves in comparison with the wild type [90,101]. Conversely, the leaf aspect ratio of the *smxl6/7/8* triple mutant was increased, and the *max2* leaf phenotypes were restored by mutation of *SMXL6/7/8*. Although no effect on the petiole of KAR2 and SMAX1 was detected, reduced blade width and length were observed in *smax1max2* and *smax1smxl6,7max2* mutant plants compared with *max2* or *smxl6,7max2* mutant plants. The phenotypic analysis of Arabidopsis leaves suggested that *smxl6/7/8*, *smax1* and their respective receptor mutants may perform opposite functions in leaf morphology [28,90].

### 4.4. Shoot Branching

There are many pieces of evidence for the involvement of SLs in branching. SL-related mutants exhibit an increased branching or tillering phenotype. For example, the high-branching/tillering phenotypes of SL synthetic mutants (*max1*, *max3*, and *max4*; *dad1* and *dad3*; *rms1* and *rms5*; *d10*, *d17/htd1* and *d27*) are restored by the exogenous application of SLs. However, the phenotypes of SL response mutants (*max2*, *dad2*, *rms4*, *d3* and *d14*) could not be restored with SL application [29,102,103]. Recently, exogenous SLs were shown to inhibit outgrowth of axillary buds in apple (*Malus spectabilis*) [104]. SLs secreted by roots suppress plant branching mainly through upward transport to axillary buds [105]. In rice, mutation of D53, which is normally a repressor of the SL signal pathway, is a gain-of-function mutant with a multi-branching phenotype. A triple mutant of SMXL6/7/8, homologous proteins of D53 in Arabidopsis, exhibited a reduced branching phenotype. In addition, the multi-branched phenotype of *max2* can be restored in a *smxl6/7/8* mutant background, suggesting that D53-like SMXLs regulate shoot branching in a MAX2-dependent manner [89,90]. IPA1 (ideal plant architecture 1) acts as a targeted transcription factor downstream of D53 and is involved in the regulation of SL-mediated tillering and D53 expression in rice [106].

SLs also regulate the development of bryophytes, such as promoting spore germination and branching [107]. In *Petunia hybrida*, an ABC protein PaPDR1 (*Petunia axillaris* PLEIOTROPIC DRUG RESISTANCE 1) acts as a transporter of SLs and regulates symbiotic signaling and branching. Increased branching and reduced symbiotic interactions were observed in the *pdr1* mutant, which was caused by impaired SL allocation due to defective SL exudation [108]. NtPDR6 (*Nicotiana tabacum* PLEIOTROPIC DRUG RESISTANCE 6) is a homologous protein of PaPDR1 and plays a key role in regulating plant branching [109], indicating that the transport of SLs may be similar in different plants.

The regulation of branching by SLs first involves the interaction with other plant hormones (see the following hormone section for details). Secondly, SLs influence the branching of plants by regulating the branching-related genes, including the TCP transcription factors BRANCH1 (BRC1) in tomato, Arabidopsis, pea and potato, FINECULM1 (FC1) in rice, and TEOSINTE BRANCHED1 (TB1) in teosinte and maize [110,111,112,113,114]. SLs also play important regulatory roles in secondary growth of dicotyledonous plants. SL promotes stem secondary growth by affecting cambial cell division and inducing specific genes. For instance, expression of the *MAX2* gene under a cambium-specific (*WOX4*) promoter could restore the secondary growth defect of the *max2* mutant [115]. More recently, a study reported that SL is required for shoot elongation by its mediation of gibberellin metabolism and signaling in rice [116].

To date, there is no evidence that KARs can regulate the branching of plants. Any discovery of KAR signaling-related mutants may help to explore whether KAR functions in regulating plant branching.

### 4.5. Root System Development

Numerous studies have revealed that SLs are involved in the regulation of root development. SLs promote the elongation of the primary root and root hairs and inhibit the formation of the lateral root in Arabidopsis [33,117]. For example, a shortened taproot was observed in the SL-deficient *ccd8* mutant in pea seedlings. In rice SL-deficient mutant, the distinct root-cap was reduced, which could be restored by application of SLs [118,119]. SLs also inhibit adventitious root formation in Arabidopsis, tomato, and pea [120,121,122]. Recent research shows that SLs induce the presence of hypodermal passage cells (HPC) in Petunia roots, which may play a role in regulating water and nutrient exchange between the root and the soil. Further study showed that this process depends on the KAI2/MAX2 pathway, suggesting that SLs and KARs play roles in the appearance of HPC [123].

A recent study demonstrated that KAI2 signaling is an important regulator of root hair and root development in Arabidopsis, mainly through the KAI2-SMAX1/2 pathway [124]. Interestingly, SL and KAR signaling together control the density of lateral roots, while most root traits, including root growth direction, root straightness and root hair development, are determined by KAR signaling alone. This finding provides new evidence for dissecting the different roles of SL and KAR signaling in root and root hair development.

### 4.6. Mycorrhizal Symbiosis

Most terrestrial plants can form symbiotic relationships with arbuscular mycorrhiza. SLs are rhizosphere signals that are recognized by AM, which lead to the germination of spores and the formation of mycelial branches [35,125,126]. One of the most important functions of SLs is this promotion of symbiosis with AM. The synthetic SL GR24 is used as a root drench to promote the difficult colonization with arbuscular mycorrhiza and eventual formation of symbiosis [108,127]. GR24 enhances mycorrhizal colonization in rice, petunia, and pea [29,128]. After arbuscular mycorrhizal colonization, the level of SLs decreased in tomato [129]. Similarly, the colonization of pea by arbuscular mycorrhizal resulted in a decrease in the germination rate of parasitic seeds due to a decrease in the content of SLs [130]. Moreover, arbuscular mycorrhizal colonization may inhibit the formation of legume nodules, due to the decrease in SL content [131]. For example, the root exudates of the SL-deficient *rms1* mutant in pea have lower SL content and reduced nodulation capacity compared with the wild type, suggesting that SLs, as positive regulators of nodulation in peas, are necessary for optimal nodulation [118]. A recent study shows that GmMAX2-mediated SL and KAR signaling are involved in regulating soybean–rhizobia interaction and nodulation through interactions with auxin and JA hormones [132].

In Arabidopsis, KARs induced seed germination, inhibited hypocotyl elongation, and promoted cotyledon opening. The germination of a KAR mutant was not alleviated by application of KARs [56]. DWARF14-LIKE (D14L) is homologous to KAI2 and co-participates in the regulation of MAX2-dependent KAR signaling pathways [91,133]. The *hebiba* mutant lost its ability to respond to arbuscular mycorrhizal fungi in rice, and D14L may be responsible for this loss of symbiosis, suggesting that KAR receptor complexes are involved in the perception of arbuscular mycorrhizal fungi in rice [134].

While SLs and KARs have similar molecular structures and produce similar effects in some ways, plant responses to SLs and KARs also differ in several aspects. For instance, both GR24 and KARs inhibit the light-regulated elongation of the hypocotyl [99]. The inhibition of hypocotyl elongation by MAX2- and light-dependent SL signals is mainly achieved by cryptochrome and phytochrome signaling pathways, among which the regulatory factors mainly include COP1, HY5, and PIFs [135]. On the other hand, SLs promote the germination of parasitic seeds, while KARs cannot, but KAR has a stronger influence on non-parasitic plant seed germination than GR24 [83,136]. In Arabidopsis, D14 mainly affects branch formation, leaf morphological development and root development of mature plants, but has no effect on seed germination [133,137]. However, KAI2 regulates seed germination, hypocotyl growth, and cotyledon opening, but does not play a significant role in plant branching [133]. In addition to the D14 and KAI2 proteins, there is a D14-like protein, DLK2, but it does not respond to any of the SL or KAR signals [133].

## 5. Hormone Interactions During SL- or KAR-Mediated Plant Development

Recent studies have shown that SLs can cooperate with or antagonize other plant hormones during plant development and biotic and abiotic stresses. Similarly, KARs may work with many plant hormones (Figure 3 and Figure 4, Table 1).

### 5.1. SL or KAR Crosstalk with Auxin

Auxin is a key factor in regulating plant growth and development. Auxin is synthesized mainly at the tops of branches and in young leaves and is transported downward in the main stem by the polar auxin transport stream (PATS) [152]. The TRANSPORT INHIBITOR RESISTANT 1/AUXIN F-BOX (TIR1/AFB) protein recognizes auxin, and together they form a co-receptor complex through E3 ubiquitin ligase with Aux/IAA protein, which acts as a repressor of the transcription of auxin-regulated genes. Aux/IAA interacts with the transcription factor AUXIN RESPONSE FACTOR (ARF) and is ubiquitinated and degraded in an ARF-dependent manner [153]. During auxin signal response, TPL (TOPLESS) interacts with IAA12/BODENLOS (IAA12/BDL) through an ethylene-responsive element binding factor amphiphilic repression (EAR) motif [154]. Interestingly, D53 and SMXL6/7/8 in SL signaling also interact with TPL through the EAR motif [32,90]. This result implied that SL and auxin signaling may be integrated by TPL to co-regulate multiple aspects of plant growth and development.

Auxin regulates shoot branching by inducing the expression of *CCD7* and *CCD8* genes, which are involved in the synthesis of SLs [155]. Transcriptome analysis revealed that SLs inhibit bud growth by negatively regulating auxin transport in rice [156]. SLs regulate root development by inhibiting the transport of auxin from shoots to roots and auxin flux within root tissues, and regulate branching by affecting polar auxin transport (PAT)/canalization in rice [157,158,159]. When auxin is depleted, SL can inhibit bud outgrowth in pea. The branch number in auxin-response mutants is inhibited by exogenous application of SLs. In addition, the production of auxin-dependent SLs is the main factor inhibiting branches [155]. Additional evidence also supports this view. The inhibition of SLs in main stem branches is dependent on the presence of auxin, while SLs enhance the competitiveness between two branches located on a common stem by inhibiting polar auxin transport [160]. SLs cause the depletion of the auxin exporter PIN1 (PIN-FORMED 1) on the plasma membrane of xylem parenchyma cells in the stem, thus regulating shoot branching [161]. At the same time, it also leads to the accumulation of auxin in the cells of the primary root meristem, which promotes the growth of the taproot, mainly due to an increase in cell length and a decrease in cell diameter [33]. Furthermore, auxin is involved in the regulation of secondary growth and root hair elongation both upstream and downstream of SL, respectively [115,162].

Lower concentrations of SLs promote root hair elongation by inhibiting auxin efflux, but higher concentrations of SLs enhance auxin efflux and inhibit root hair elongation and asymmetric root growth [163]. GR24 does not directly affect the expression of PIN, but the effect of SLs is dependent on the auxin status and seems to modulate the level of auxin. The inhibition of lateral root primordium by SLs is partially mediated via a reduction in the level of auxin, and SLs reduce the levels of auxin in the leaf tissue. For instance, exogenous application of GR24 reduced the content of auxin in the leaf of the SL-deficient *max4* mutant [33]. Moreover, SLs affect the formation of lateral roots by affecting the polarity and localization of PIN proteins [159]. Recent studies have shown that PIN1-mediated polar transport of auxin is involved in the regulation of the branching process through the regulation of SLs. A number of auxin exporters, including PIN3, PIN4, and PIN7, play important roles in shoot formation. These transporters regulate a mechanism called connective auxin transport (CAT), which mediates the regulation of branching by SLs in a BRC1-independent manner [139]. Moreover, new evidence shows that sucrose represses the auxin-induced SL pathway to promote bud outgrowth [164], suggesting that sucrose and hormones (auxin and SLs) also play important roles in the regulation of bud outgrowth.

SLs also inhibit adventitious root formation in different plants, such as Arabidopsis, tomato, and pea [120,121,122], but promote the crown root growth in rice [119]. The effect of auxin on adventitious root formation is opposite to that of SLs, and SLs could partially repress the stimulating effect of auxin on adventitious root formation [120]. SLs strongly interact with auxin signaling to regulate secondary growth, and play a positive role in the pathway downstream of auxin. For example, the positive effect of auxin on cambium was reduced in the *max* mutants [115]. Altogether, auxin, as a major regulator of the synthesis of SLs, antagonizes the function of SLs by enhancing the transport of auxin [165,166,167].

The interactions between auxin and KAR signaling are still unclear. KARs reduce the level of endogenous auxin by inhibiting the expression of IAA-responsive genes. This promotes seed germination [168].

### 5.2. SL or KAR Crosstalk with Gibberellin

Gibberellic acid (GA) is an important regulator of plant growth and development. Defects in GA synthesis and signaling lead to many defective phenotypes, such as inhibited germination, delayed root growth and flowering, male sterility, dwarfing, reduced rate of seed setting, and increased tiller buds [169,170].

GA-deficient mutants have the same multi-branched phenotype as SL-deficient mutants [171]. Surprisingly, the GA signaling pathway shows the most striking similarities with the SL and KAR signaling pathways. These pathways all contain components that are degraded by the 26S proteasome. When GA is recognized by the α/β hydrolase receptor GID1 (GIBBERELLIN-INSENSITIVE DWARF 1), they form a complex which binds to DELLA proteins to form a stable trimer. Through the E3 ligase SCF^SLY1/GID2^, the DELLA proteins are ubiquitinated and degraded, removing the inhibitory effect of DELLA proteins and allowing plant growth [172,173,174]. The DELLA proteins in the GA signaling pathway have similar roles as D53-like/SMXL6, 7, and 8 proteins in SL signaling and as SMAX1 in KAR signaling. Previous studies have shown that the SL receptor D14 interacts with the GA signaling repressor SLR1 (SLENDER RICE 1) in an SL-dependent manner in rice [88]. This provides evidence for crosstalk between SLs and GA.

In general, GA participates in the regulation of seed germination by KAR signaling. KARs promote germination of dormant Arabidopsis seeds, and the stimulation of such germination is partly dependent on DELLA proteins. KAR1 can partially restore the inhibited seed germination of the GA-insensitive *sleepy1* mutant [83]. ABA may inhibit the effect of KARs on the germination of Arabidopsis seeds by down-regulating the expression of the GA synthesis gene [83]. On the other hand, KARs also inhibit the synthesis of GA by inducing the production of ABA to delay the germination of soybean seeds [100]. Therefore, the upstream and downstream relationships of KARs, ABA, and GA are not completely consistent between different plant seeds, suggesting that they play opposite roles. At the same time, KAR treatment can increase the expression of the GA_3_ oxidase gene in Arabidopsis seeds [99]. Inhibitors of GA synthesis had an inhibitory effect on seed germination induced by smoke [175]. KAR_1_ and GA_3_ synergistically inhibit ABA activity and release seed dormancy [176].

In the crosstalk between plant hormones, participation in the regulation of the same physiological process does not mean that the plant hormones are mutually dependent on each other to regulate this process. For instance, both SL and GA can affect internode elongation by stimulating cell division, but SL works independently of the GA signal in this process [177]. In addition, the roles of SL and GA in branch regulation are independent in pea [178]. GA_3_ could significantly increase seed germination of *kai2* and *max2* mutants, indicating that KAI2 and MAX2 may not be involved in the stimulation of GA on seed germination [63].

### 5.3. SL or KAR Crosstalk with Abscisic Acid

SLs and ABA are both derived from the carotenoid pathway, beginning with the transformation of 9-cis/all-trans-carotene into the precursor all-*trans*-violaxanthin [54]. The similarity of the synthetic pathways provides a sufficient and favorable basis for the interaction between SL and ABA functions. First of all, ABA and SL affect each other’s synthesis. For instance, in the ABA-deficient mutants *up14* (maize), *notabilis* and *sitiens* (tomato), the transcriptional levels of the SL synthesis genes *CCD7* and *CCD8* are reduced, which leads to decreased content of SLs in root secretion [141]. Further, SL synthesis is regulated by ABA in Arabidopsis [41]. KARs negatively regulate seed germination by inhibiting GA synthesis and promoting ABA synthesis in soybean [100]. It was also reported that the *max2* mutant has lower ABA sensitivity. In addition, the MAX2 gene was detected at high expression levels in ABA-treated seedlings, implying that MAX2, as an important component of SL signaling, also participates in the ABA signaling pathway [48,142]. During study of symbiosis, it was found that AM colonization was decreased in the ABA-deficient mutant *sitiens*, suggesting that ABA plays a role in arbuscular mycorrhizal fungi symbiosis by regulating the production of SLs [141,144]. A recent study indicated that ABA is also involved in SL-mediated dormancy of axillary buds in rice, which represents a new insight into the mechanism by which SL inhibits outgrowth of axillary buds [179]. Moreover, CYP707As, involved in abscisic acid catabolism, are effectors newly discovered to be involved in SL and KAR responses in Arabidopsis and parasitic plants [180]. However, how CYP707As link ABA and SL/KAR signaling remains unclear.

### 5.4. SL or KAR Crosstalk with Ethylene

Ethylene, a gaseous plant hormone, is involved in regulating seed germination, hypocotyl elongation, root and root hair elongation, inhibition of lateral root development, and leaf senescence [98,145]. Ethylene is produced during the germination of most seeds, and ethylene promotes seed germination in a dose-dependent manner [181,182]. SLs promote the production of ethylene [183]. These results imply that ethylene is involved in the regulation of seed germination by SLs, and that ethylene biosynthesis is essential for SL-mediated promotion of seed germination [184]. AVG, an inhibitor of ethylene biosynthesis, also inhibits seed germination induced by the SL analogue GR24, but this effect could be overcome by ACC (a reaction product of AVG inhibition) [182]. In addition, both SLs and ethylene are positive regulators that depend on a common regulatory pathway, at least in root hair elongation. In the ethylene signaling-deficient *ein2* and *etr1* mutants, the root hair elongation response to SLs is reduced. These results indicated that the synthesis of ethylene was necessary for the promotion of root hair elongation by SLs, while it is also possible that auxin signaling could be integrated with SL and ethylene signaling to regulate the elongation of root hairs [145].

The formation of adventitious roots can be regulated by both SLs and ethylene. Previous research suggested that SLs and ethylene played independent roles in adventitious root formation, but that the ethylene precursor ACC and SLs played antagonistic roles in the first third of the hypocotyl. ACC promoted adventitious root formation, while ACC and SLs inhibited adventitious root formation in the lower part of the hypocotyl [185].

SL-deficient and SL-insensitive mutants, including *max2* in Arabidopsis, *dad1* in pea and *dwarf* mutants in rice, exhibited delayed senescence. Moreover, the delayed senescence of leaves caused by the absence of SLs was inhibited by application of SLs [75,101,146]. Ethylene is also involved in the regulation of leaf senescence. Ethylene treatment significantly induced the expression of the SL synthesis genes *MAX3* and *MAX4*, indicating that SLs can be synthesized in senescent leaves. At the same time, exogenous SL application in the presence of ethylene enhanced the promotion of ethylene on leaf senescence [34].

### 5.5. SL or KAR Crosstalk with Cytokinin

Cytokinins (CKs) are plant hormones that can either cooperate with or antagonize the action of SLs in regulating plant growth and development. For example, SLs inhibit the elongation of rice mesocotyl cells, while CKs promote it [186]. The antagonism between CKs and SLs was also demonstrated in the regulation of bud outgrowth in pea [147]. In rice, the branching related gene *FC1* (*FINECULM1*) is insensitive to SLs, but is inhibited by CKs [148]. The expression of *BRC1*, a homolog of *FC1* in pea, is promoted by SLs and inhibited by CKs [112,147]. Recent studies have shown that SL promotes the degradation of CKs through activating the expression of *CKX9* (*CYTOKININ OXIDASE 9*) in rice, which encodes an oxidase that catalyzes the degradation of CK [149]. In rice *d53* mutants, the CK content in the stem base is significantly increased. The expression of the CK catabolic enzyme *OsCKX9* is induced by GR24 in wild type but not in *d53* mutant. Moreover, *OsCKX9* responds to SLs and plays a role in the SL signaling pathway [149]. In these ways, SLs and CKs are involved in regulating shoot architecture in rice.

The SL-response mutant *max2* shows decreased sensitivity to the synthetic CK 6-Benzylaminopurine (BAP) [187]. SLs inhibit the formation of lateral roots in a MAX2-dependent manner. The CK signaling components AHK3 (HISTIDINE KINASE 3), ARR1 (RESPONSE REGULATOR 1), and ARR12 (RESPONSE REGULATOR 12) are involved in the effects of GR24 on lateral root development. SLs also influence the formation of lateral roots by affecting the polarity of auxin [150,159]. Therefore, the development of lateral roots is accompanied by crosstalk among SLs, auxin, and CKs. Interestingly, the inhibition of adventitious roots by SLs is independent of CK [120]. CK is also involved in the regulation of seed germination, and the SL analogue GR24 promotes increases in the content of CK in Striga seeds, indicating that SLs are an upstream signal of CK during the regulation of seed germination [97].

The TMB can inhibit the germination of lettuce seeds. On the one hand, it increases ABA content through a photosensitive pigment system, on the other hand, it inhibits cytokinin homeostasis under dark conditions [100]. Another evidence that KAR is involved in regulating CK is that KAR_1_ and SW are able to raise the levels of primary endogenous cytokinins in *Eucomis autumnalis* and *Spinacia oleracea* L plants. For example, SW and KAR_1_ increase significantly the levels of ciszeatin, dihydrozeatin, and isopentenyladenine of CKs and then yield a greater number of leaves in spinach plants [188]. Meanwhile, SW and KAR_1_ treatments also accumulate higher concentrations of isoprenoid-type CKs in the aerial organs of *Eucomis autumnalis* [189]. These data indicate that the crosstalk between KAR and CK also plays an important role in plant growth and development.

### 5.6. SL or KAR Crosstalk with Other Hormones

SLs can also interact with other plant hormones to regulate plant growth and development. It was found that MAX2 interacts with BES1 (*bri1*-EMS-suppressor 1), a positive regulator in the brassinosteroid signaling pathway, to accelerate the degradation of BES1 in the presence of SLs [151]. Recent study showed that the contents of free phenolic acids and salicylic acids were increased significantly in spinach plants treated with SW and KAR_1_, implying that there is a crosstalk possibility between KAR and salicylic acid [188].

## 6. SLs and KARs During Abiotic Stress: Responses and Adaptation

### 6.1. Dynamic Regulation of SLs Under Abiotic Stresses

In recent years, substantial evidence has demonstrated that SLs and KARs are involved in the regulation of the plant responses to abiotic stress [8,37,38,41,42,43,46,63,143,190,191] (Table 1). SL levels are finely modulated under various types of abiotic stress. Recent studies have shown that SL biosynthesis is repressed in the roots of tomato plants under drought stress conditions. Moreover, a drop in the biosynthesis of SL in roots during drought may act as a systemic signal affecting SL synthesis in aboveground organs [38,143]. Interestingly, drought increases the abundance of transcripts of the SL-biosynthetic genes *SlCCD7* and *SlCCD8* in tomato shoots and the *D27* and *MAX1* homologs *Os01g0700900*, *Os01g0701400*, *Os02g0221900*, and *Os06g0565100* in rice shoots [38,40]. Additionally, the SL-biosynthetic genes *MAX3* and *MAX4* are significantly induced by dehydration and salinity in leaves of Arabidopsis [41]. Essentially, these findings suggest that an efficient activation of SL biosynthesis is triggered in response to osmotic stress, leading to the activation of SL signaling, which positively regulates the tolerance of these adverse conditions.

### 6.2. SL- and KAR-Mediated Plant Adaptation to Abiotic Stresses

Numerous loss-of-function and exogenous SL treatment studies have revealed that SLs contribute to the responses to drought and salinity in Arabidopsis, rice, and rapeseed [40,41,42]. In Arabidopsis, hypersensitivity to drought and salt stress was observed in mutants of both the SL-biosynthetic genes *MAX3* and *MAX4* and the SL-signaling gene *MAX2* [41]. Additionally, transcriptome analysis of *max2* leaves revealed that genes related to drought and ABA responses are downregulated. On the other hand, photosynthetic genes, which are generally repressed under dehydration in an ABA-independent manner, are upregulated in *max2* compared to wild type (WT) leaves under normal and drought conditions [41]. This implies that MAX2 regulates the drought stress response in both an ABA-dependent and ABA-independent manner.

Spraying plants with SL confirmed the role of SL as a positive regulator in stress responses. In grapevine, foliar application of GR24 could alleviate drought stress by regulation of stomatal closure and photosynthesis and activation of antioxidant defense [50]. SL-deficient *Lotus japonicas Ljccd7-* and tomato *SIccd7*-silenced transgenic plants also exhibit sensitivity to osmotic stress [38,143]. Interestingly, research on Arabidopsis and tomato indicates that SLs contribute to ABA-mediated stomatal closure under drought stress [38,41]. Recently, SLs were reported to contribute to the triggering of stomatal closure by stimulating the production of H_2_O_2_ and NO. This implies that SL signaling is linked to H_2_O_2_ and NO signaling in adverse conditions [37]. MAX2 functions in two pathways: D14-mediated SL signaling and KAI2-mediated KAR/KL signaling. The drought sensitivity of *max2* may be attributed to defects in the signaling pathways of SL, KAR/KL, or both. A recent study has shown that the KAR/KL receptor KAI2 positively regulates tolerance to drought stress by enhancing cuticle formation, stomatal closure, cell membrane integrity and anthocyanin biosynthesis in Arabidopsis [8]. These findings suggest that the KAR/KL-mediated signaling pathway also contributes to the improvement of drought tolerance in plants. Additionally, Li et al. (2017) reported that *d14* mutant plants are more sensitive to drought stress compared to wild type, indicating that the SL receptor D14 is also involved in drought responses [8]. Interestingly, *d14kai2* double mutant plants are more sensitive to drought stress than the *d14* and *kai2* single mutants [8], suggesting that the D14-mediated SL pathway and the KAI2-mediated KAR pathway act together to enhance tolerance to drought stress in Arabidopsis.

The *max2* and *kai2* mutants exhibit decreased cuticle thickness and enhanced cuticular water permeability, implying that SL and/or KAR signaling pathways are involved in regulating cuticle thickness [8,48]. In contrast, the *d14* mutant does not exhibit cuticle defects, unlike the *kai2* mutant and the *d14kai2* double mutant [8]. This implies that a D14- or KAI2-dependent signaling component may exist as a distinct mechanism in adaptation to drought in Arabidopsis. Recently, Wang et al. (2018) reported that the germination of *kai2* seeds is more sensitive to osmotic stress, salinity, and high temperatures [63]. Interestingly, KAR-induced KAI2 signaling promotes germination under favorable conditions and inhibits germination under unfavorable conditions in Arabidopsis seeds. This suggests that KAI2-mediated signaling may play an important role in responses to abiotic stress by maintaining viability while inhibiting germination under unfavorable conditions [63].

Nitrogen (N) and phosphate (P) are key nutrients required for plant growth. A deficiency in these nutrients critically affects the sustainable production of crops [192]. Several studies have reported that SLs are involved in the regulation of root development under P- or N-deficient conditions. In rice, the *d10* and *d27* mutants in SL-synthesis and the *d3* mutant in SL-signaling decreased seminal root density and increased lateral root density during P or N starvation compared to wild type. In addition, the exogenous application of GR24 restored the reduced response to low-P or low-N conditions in the *d10* and *d27* mutants, suggesting that SLs promote seminal root development and negatively regulate lateral root development under nutrient stress [157,193]. In Arabidopsis, SL-deficient *max4* and SL-response *max2* mutants have shorter root hair lengths under low-P conditions compared with wild type. This defective phenotype can also be restored with GR24 in the *max4* mutant [47]. Collectively, SLs may trigger the fine-tuning of root architectures via the MAX2 component of SL signaling under unfavorable nutritional conditions to promote plant adaptation to adverse environments.

SLs have recently been reported to positively regulate chilling tolerance in pea plants and Arabidopsis [43]. In pea, biomass accumulation was decreased in the SL-signaling mutant *ramosus3* (*rms3*) and the SL-synthesis mutant *rms5* after chilling in the dark. Similar results were also observed for the SL-synthesis *max4* mutant in Arabidopsis [43]. Also under dark chilling, photosynthetic carbon assimilation was inhibited in *rms* mutants in pea and in *max3*, *max4*, and *max2* mutants in Arabidopsis [43]. These finding suggest that SLs play a role in the dark chilling tolerance of photosynthesis in pea plants and Arabidopsis. In addition, a significant reduction in leaf area has been observed following dark chilling treatment in the presence of GR24 [43], implying that SL signaling may help plants adapt to a low temperature environment by regulating the growth of leaves under chilling stress. However, this needs to be clarified in the future studies.

**Figure 5:**
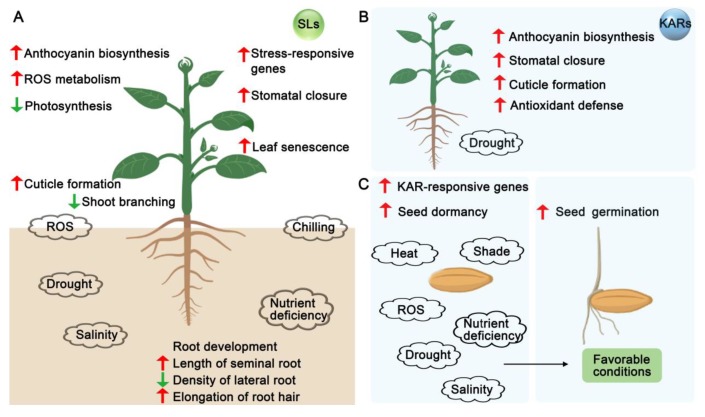
Model for the involvement of SLs and KARs in various abiotic stress responses. (**A**) Increasing evidences suggest that SLs are involved in plant adaptation to abiotic stresses (e.g., drought, salinity, nutrient deficiency, chilling, and oxidative stress). SLs can fine-tune root development by increasing the length of seminal roots and root hairs and decreasing the density of lateral roots. Aboveground, elevated levels of SLs in shoots may positively regulate stomatal closure, cuticle formation, and stress-responsive genes to reduce water loss. SLs may also be involved in accelerating leaf senescence by activating ethylene-mediated senescence signaling, which may further activate the process of nutrient relocation under abiotic stresses. In addition, SLs may also inhibit shoot branching and photosynthesis to optimize plant adaptation to stress. SLs are also involved in regulating anthocyanin biosynthesis to alleviate oxidative stress induced by various abiotic stresses. (**B**) KARs contribute to the protection against abiotic stress during seed germination and promote drought resistance in Arabidopsis. (**C**) KARs-KAI2 signaling can maintain seed dormancy and inhibit germination under abiotic stress and can stimulate seed germination under favorable conditions. Upward red arrows indicate a positive response, and downward green arrows indicate a negative response. SLs, strigolactones; KARs, karrikins; ROS, reactive oxygen species.

Several flavonoid biosynthesis-related genes are significantly repressed by dehydration stress treatments in both *max2* and *kai2* mutants compared with those in wild type. This implies that SL and/or KAR can positively regulate flavonoid biosynthesis under drought stress [8,41]. Indeed, a lower level of anthocyanin has been observed in *kai2* mutant plants compared with wild type under water-deficit conditions [8]. In addition, the abundance of several flavonol biosynthesis-related enzymes are significantly reduced in the *max2* mutant, while GR24 is able to induce flavonol accumulation in a MAX2-dependent manner [194]. It is well known that flavonoids/anthocyanins protect plant tissue from various environmental stresses [195,196,197]. These results suggest that the SL and KAR signaling pathways are able to stimulate anthocyanin accumulation under adverse conditions, which may contribute to drought tolerance in plants (Figure 5).

## 7. SLs and KARs Crosstalk with Other Phytohormones Under Abiotic Stresses

Several studies have provided evidence for the interaction of SL or KAR signaling with the signaling of other hormones during responses to biotic and abiotic stress (Table 2) [40,41,52,137,165,167,191,198,199,200,201]. Both SLs and ABA biosynthetically originate from carotenoids [202]. Reduced SL levels in ABA-biosynthesis mutants have been observed in tomato plants [141,203], while SL-deficient tomato mutants exhibited a reduction in the levels of ABA [204]. This suggests that ABA and SL interact with each other during biosynthesis. These results agree with the observed reduction of ABA levels under low-P and osmotic stress conditions for SL-deficient CCD7RNAi lotus plants [143], implying that SL deficiency reduces ABA levels. Moreover, it has been reported that SL signaling-mediated adaptation to drought stress is associated with ABA-mediated stomatal closure [38,41]. In addition, exogenous GR24 pretreatment may increase stomata sensitivity to ABA in tomato plants [38]. In Arabidopsis, drought-susceptible *max2* plants exhibited impaired ABA-mediated stomatal closure [41,48], yet no differences in ABA contents have been observed between the wild type and *max2* following drought treatment [48]. Additional evidence has demonstrated that SL positively regulates the stress and ABA signaling pathways by regulating the expression of many stress and/or ABA-responsive genes, which are involved in abiotic stress response [41]. These findings suggest that SL-promoted drought tolerance may be a partially ABA-dependent pathway in plants. However, in rice, the SL biosynthetic *dwarf10* (*d10*) and *d17* mutants and the SL perception *d3* mutant exhibit an increased ABA concentration under normal and drought conditions and enhanced drought tolerance when compared with wild type [40]. In contrast, the mutation of *D27*, which encodes the initial enzyme in SL biosynthesis and involves the conversion of all-*trans*-β-carotene to 9-*cis*-β-carotene, decreased the ABA levels and drought tolerance [40], while overexpression of OsD27 increased the levels of ABA. These results demonstrate that SL signaling is linked to the ABA pathways through D27, which plays an important role in determining the ABA and SL content in rice. These contradictory observations, of ABA levels in rice SL mutants on the one hand and Arabidopsis, tomato, and lotus on the other hand, may be due to differences in monocots and dicots. The different mechanisms could be revealed by performing comparative genome-wide expression profiling studies among these plant species.

A recent study has reported that *kai2* mutant plants exhibit a decreased sensitivity to ABA, suggesting that KAI2 positively regulates the ABA response [8]. In addition, *kai2* mutants exhibit increased ABA concentrations compared to wild type under both well-watered and dehydration conditions. This may due to the downregulated ABA catabolic gene *CYP707A3* and ABA transport-related genes *ABCG40* and *ABCG22* in *kai2* when compared with wild type [8]. A recent study demonstrated that the CYP707A protein, which is involved in ABA catabolism, may act as an effector of the KAR and SL signaling pathways in Arabidopsis and parasitic plants [180]. These findings provide evidence for the involvement of the crosstalk between ABA- and KAI2-dependent signaling pathways in plant adaptation to drought. Interestingly, ABA-mediated stomatal closure and the reduced sensitivity to ABA may be common strategies in SL and KAI signaling for adaptation to drought [8,41,48]. The mechanisms of SL and/or KAR signaling in regulating ABA-mediated stomatal movement need to be further clarified in the future.

In Arabidopsis, transcriptome analysis has revealed that the four CK catabolism-related genes *cytokinin oxidase 1* (*CKX1*), *CKX2*, *CKX3*, and *CKX5* were downregulated in the *max2* mutant prior to and during dehydration [41]. Previous studies have reported that the overexpression of *CKX1*, *CKX2*, *CKX3*, and *CKX4* increased responses to ABA and improved the tolerance to drought and salt in Arabidopsis and tobacco [208,209]. The CK signaling pathway has been shown to negatively regulate tolerance to drought and salt stress by antagonizing ABA signaling [13,209]. These findings suggest that SL signaling and/or KAR signaling may negatively regulate CKX-mediated CK levels during dehydration, which subsequently contribute to the modulation of stress tolerance by influencing the ABA response. Although CKs and SLs have been reported to antagonistically regulate bud activation and shoot branching [147,166] and to synergistically regulate lateral root development [150], direct evidence of the interaction between SL signaling and CK signaling under abiotic stress is lacking [210]. The hypothesis that SL and/or KAR signaling positively regulate abiotic stress tolerance in plants by reducing CK contents and/or inhibiting CK signaling remains to be further demonstrated. In addition, studies on the CK/SL biosynthesis mutants and CK/SL signaling mutants under drought conditions will contribute to the revealing of the potential crosstalk between the SL and CK signaling pathways in plant adaptation to drought stress.

A recent study has provided evidence that GA and the synthetic SL rac-GR24 result in predominantly additive transcriptional changes of a largely overlapping set of genes [201]. A previous study reported that SLs alleviate seed thermoinhibition by modulating the ABA/GA ratio via decreasing ABA levels and increasing GA levels in Arabidopsis, suggesting that SL may act upstream of ABA and GA to regulate seed thermoinhibition [97]. Furthermore, the application of exogenous GA_3_ stimulates seed germination of wild type, the SL-related *d14* and *max2* mutants, and the KAR-related *kai2* mutant in both the absence and presence of NaCl in Arabidopsis [63]. This finding implies that the promotion of seed germination by GA_3_ is independent of the D14-MAX2 or KAI2-MAX2 signaling pathways in Arabidopsis. Additionally, GA negatively affects SL biosynthesis by regulating the expression of SL biosynthesis genes in rice [140]. Moreover, application of exogenous GA reduced the infection of rice by the parasitic plant Striga [140]. These results suggest that crosstalk between the GA and SL signaling pathways may be an advantage for the management of root parasitic weeds.

In Arabidopsis, the SL-biosynthesis mutants *max1*, *max3*, and *max4* and the SL-response mutants *d14* and *max2* exhibited a delay in leaf senescence during dark and ethylene treatments. However, this was not the case for the KAR-response mutant *kai2* [34]. Moreover, *MAX3* and *MAX4*, which are important SL biosynthesis genes, were strongly induced during dark and ethylene treatments [34]. In addition, leaf senescence was triggered by the application of GR24 only in the presence of ethylene and not by GR24 alone. These findings suggest that SLs are involved in the acceleration of leaf senescence by activating ethylene-mediated senescence signaling [34]. It is well known that leaf senescence is an active process of nutrient relocation, which is beneficial for recycling nutrient materials from dispensable leaves [210]. Although the underlying mechanism of the interaction between SL and ethylene under abiotic stress conditions is unclear, we hypothesize that the crosstalk between ethylene and SL may have the ability to accurately regulate leaf senescence under adverse environmental conditions. Future studies will be required to fully understand crosstalk between ethylene and SL signaling in processes such as leaf senescence and adaptation to abiotic stress, and how SL signaling components activate ethylene signaling under normal and adverse conditions.

Several studies have revealed that the crosstalk between auxin and SL signaling is the predominant contributor to the regulation of root development under nutrient shortages [145,157]. Experimental data in rice suggest that SLs can fine-tune root architectures by modulating the transport of auxin from shoots to roots under N and P limitation, which in turn increases the seminal root length and decreases lateral root density. In addition, the additive effect on root hair elongation has been reported for SLs and auxin [145]. Furthermore, ethylene was also involved in the SL–auxin crosstalk during the elongation of root hairs. Here, ethylene may be epistatic to SLs, acting as a crosstalk junction between them [145]. Taken together, SLs and KARs play essential roles in plant abiotic responses, and they interact with other hormones, including ABA, CK, GA, ethylene, and auxin, to cooperatively regulate the adaptation of plants to abiotic stress.

## 8. Conclusions and Perspectives

Significant progress has been made towards an understanding of how the SL and KAR signaling pathways influence plant developmental and environmental responses. The majority of recent studies have focused on the biosynthesis and perception of SLs and KARs. However, studies on SLs and KARs in plant adaptation to environmental stresses are still at the basic stage. The involvement of SLs in regulating the responses to drought, salinity, and nutrient deficiency stress as well as chilling tolerance has been demonstrated [38,40,41,42,43,47,48,157,193]. KARs are also involved in the regulation of drought tolerance and provide seeds with abiotic stress tolerance in Arabidopsis [8,63].

However, the downstream targets of SL and/or KAR signaling pathways are not fully understood under normal or adverse environmental conditions, and the signal transduction pathway of KAR is still unclear. Moreover, there are still many outstanding questions that remain to be studied in depth. How can SLs and KARs be differentiated and regulate different signaling pathways in a MAX2-dependent manner in plant growth and development processes? What is the basis for the degradation of SMXL family proteins by ubiquitination and the 26S proteasome in different signaling pathways? What are the roles of SMXL1 and SMXL6, 7, 8, and how quickly do they perceive the signals of SL and KAR? Do SMXL family members directly regulate downstream transcription factors or indirectly through other proteins? All these questions indicate that there is still work to do to identify components of the SL and/or KAR signaling pathways and to investigate the functions of these members (e.g., SL-receptor AtD14 and the KAR-receptor KAI2). It is critical to determine the core components (e.g., MAX2, SMAX1/2, and D53/SMXLs), the yet-unknown components, and the shared components. In particular, SMAX1-LIKE/D53 family members act as transcriptional repressors of SL and KAR signaling to regulate shoot development [32,90]. Studies on the involvement of these components in plant abiotic stress, their partners and downstream transcriptional targets will further help elucidate the common or specific mechanisms of SL and KAR signaling under environmental stress. Although SLs and KARs are both butenolide molecules, plants may distinctly perceive the diverse endogenous SL and KAR/KL molecules in order to trigger optimal developmental and environmental responses. However, the endogenous KAI2-ligand remains undetected, while determining it will provide important clues for understanding KAR signaling. In addition, the production of natural SLs is limited, while their natural structures are complex and diverse. Therefore, simple and efficient detection and synthesis technologies will promote SL biological research and application, particularly in agricultural production.

SL and KAR signaling pathways may be positively involved in the regulation of flavonoid/anthocyanin syntheses in an MAX2-dependent manner under drought stress [7,40,193]. This suggests that SL and KAR signaling pathways may link the signaling pathways of reactive oxygen species (ROS) in plant responses to environmental stresses. ROS signaling has been shown to be an integral part of the abiotic stress-response mechanism, so, it remains to be determined if there is crosstalk between SL and ROS signaling under adverse environmental conditions. This crosstalk might be one approach to improving plant tolerance of abiotic stress.

SLs and/or KARs are able to link other hormone pathways and may form a regulatory network for various aspects of plant development and adaptation to abiotic stress. However, any crosstalk between SLs/KARs and salicylic acid (SA) and SLs/KARs and jasmonate (JA) has not been established due to limited experimental data. The interaction between SLs/KARs and other hormones under abiotic stress must be further explored using physiological, biochemical, genetic, and molecular biological methods. This work will clarify this complex regulatory network. Furthermore, comparative analyses using transcriptomics, proteomics, metabolomics, and functional genomics promise to add insights into the SL/KAR regulatory network. The use of CRISPR-Cas9-mediated genetic manipulation of SL and KAR signaling pathways also should provide new revelations about the molecular mechanisms by which SLs and KARs influence plant development and adaptation to stress and may provide valuable resources for crop breeding.

## Figures and Tables

**Table 1:** Mutations of strigolactone (SL) and karrikin (KAR)-related genes that alter the effects in the growth and development of various plants species.

Species	Mutants	Effects of Mutant	Functions	Interactions with Phytohormones	References
Rice	*d3, d14 and d53*	Increased branching	D53 acts as a repressor of the SL signaling to promoting axillary bud outgrowth		[89]
pea	*ccd8*		Endogenous SLs inhibit shoot branching in plants		[102]
pea	*ramosus (rms)*		SLs regulate shoot branching		[76]
Arabidopsis	*max4*				
petunia	*dad*				[138]
	*ipa1*	Regulated tiller number	IPA1 interacts with D53 to mediate tiller regulated by SL		[106]
Arabidopsis	*max1 and max2*	Increased branching, round leaves, elongated hypocotyl	MAX1 and MAX2 control shoot branching by repressing primordia formation of the axillary meristem		[101]
	*smxl6, smxl7, smxl8*	Reduced shoot branching in *smxl6/7/8*	SMXL6, SMXL7, and SMXL8 promote shoot branching by repressing BRC1/TCP18 expression in axillary buds	Auxin	[90]
		Reduced auxin transport in *smxl6/7/8*	SMXL6, SMXL7, and SMXL8 promote auxin transport in a MAX2-dependent manner		
		Lower lateral root density in *smxl6/7/8* Reduced auxin transport in *smxl6/7/8*	SMXL6, SMXL7, and SMXL8 promote lateral root density		
	*smxl6, smxl7, smxl8, max2, smax1, d14 and kai2*	In short day: elongated petiole in *smxl6/7/8,* shortened petiole in *max2* and *d14,* increased both blade length and width in *kai2*	SMAX1 and SMXL6,7,8 regulate the complementary aspects of leaf morphology in different signaling pathways		
petunia	*pdr1*	Increased branching	PaPDR1 acts as a transporter of SL to regulate branching		[108]
	*dad1/Phccd8*	Increased branching	Mutations of *PhCCD8* caused a high branching of *dad1*		[75]
		Smaller flowers	Loss of *Dad1* reduces the overall height of the plant, root and flowering development		
		Reduced internode length			
		Reduced root growth			
Arabidopsis	*ore9/max2*	Delayed senescence of leaves	Dad1/PhCCD8 and ORE9/MAX2 regulate the leaf senescence by affecting the same signaling pathway		[101]
Rice	*d17 and D10*		SLs affect leaf senescence		
Arabidopsis	*max1, max2, pin1 and pin3*	Reduced cambium activity	SLs stimulate the secondary growth in auxin-dependent	Auxin	[115]
Arabidopsis	*max4*	Reduced auxin content in the leaf	SLs reduce the content of auxin	Auxin	[33]
Arabidopsis	*max2, max4 and pin3pin4pin7*	*pin3/4/7* was restored high branching of *max2* and *max4*	PIN3, 4, and 5 of CAT contribute to branching mediated by SL	Auxin	[139]
Arabidopsis	*max1-4, ipt1,5,7 and ahk3,4*	Increased adventitious roots	SLs suppress adventitious root formation, SLs could partially restore the stimulating effect of auxin on adventitious root formation	Auxin	[120]
Pea	*rms1, rms4 and rms5*		SLs also suppress the adventitious root by reducing the size of rooting zone in Pea		
Rice	*d3, d10, d14,*	Higher epi-5DS levels by feedback relationship of SL pathway	GA_3_ regulates SL biosynthesis in a D3 and D14 independent manner	GA	[140]
	*slr1-5, gid1-3, and gid2-2*	Reduced the levels of SLs	GAs negatively regulates the level of SLs in a GID1- and GID2-dependent manner		
Species	Mutants	Effects of mutant	Functions	Interactions with phytohormones	References
Maize	*up14*	Reduced content of SL in root secretion	ABA and SL affect each other’s synthesis	ABA	[141]
tomato	*notabilis and sitiens*				
Arabidopsis	*max*	Lower sensitivity to ABA	The synthesis of SL is regulated by ABA		[41]
Arabidopsis	*max1 and max2*	Reduced seed germination	Application of GR24 can restore thermoinhibition in *max1* and *max2* caused by ABA inhibition of GA synthesis and signal and increase GA_4_ content	ABA and GA	[97]
	*max2*	Lower sensitivity to ABA	MAX2 participates in ABA signaling pathway as an important component of SL signaling pathway	ABA	[142]
*Lotus japonicus*	*ljccd7*	Decreased ABA content	SLs interaction with ABA to regulate the abiotic stress	ABA	[143]
tomato	*sitiens*	Decreased AM colonization	ABA plays a role in arbuscular mycorrhizal fungi symbiosis by regulating the production of SLs	ABA	[144]
*Physcomitrella*	*ccd8*	Increased pore germination	SLs inhibits the germination of *Physcomitrella* pore germination		[98]
Arabidopsis	*max2, ein 2-1 and etr1-1*	Reduced root hair	SL’s effect on RH elongation is dependent on both auxin and ethylene signaling	Ethylene and Auxin	[145]
	*tir1, arf7arf19 and aux1*	Increased root hair elongation			
Arabidopsis	*max2*	Delayed senescence	SL interacts with ethylene to regulate leaf senescence	Ethylene	[101]
Pea	*dad1/Phccd8*				[75]
Rice	*dwarf*				[146]
Pea	*rms1*	*rms1* are more sensitive to CK	CKs and SLs contribute to bud outgrowth in pea	CK	[147]
Rice	*fc1, d10, d3*	Increased tillering	The branching related gene FC1 (FINECULM1) is insensitive to SLs, but is inhibited by CK	CK	[148]
Rice	*d53*	Increased CK content	SLs promote CK degradation	CK	[149]
Arabidopsis	*ahk3, arr1 and arr12*	Lateral root development insensitive to GR24 and affected polar auxin transport	SLs connects with auxins and CKs to regulate LR development	CK and Auxin	[150]
Arabidopsis	*max2 and bes1*	Enhanced rosette branching	MAX2 interacts with BES1 to regulate branching	BR	[151]
Arabidopsis	*hy5*	Shortened hypocotyl	KARs restore the hypocotyl elongation inhibited by red light in *hy5*		[99]
	*ga1*	KAR1 cannot promote the germination of *ga1-3* of GA synthesis defect	KAR1 promotes germination is required for GA biosynthesis	GA	[83]
	*sleepy1*	Delay seed germination	KAR1 promotes germination is partly dependent on DELLA		
Soybean	Wild type		KARs delay seed germination under shaded conditions by inhibiting GA synthesis and promoting ABA synthesis	GA and ABA	[100]
Arabidopsis	*kai2, kai2/d14*	Decreased root hair density; Exaggerated skewing and waving	KAI2 signaling pathway regulated root hair and root development		[124]
*Lactuca sativa*	Wild type	Smoke-water and KAR_1_ promote seed germination	Application of smoke-water and KAR_1_ decrease ABA content and enhance hydrolase activity to mobilize stored reserves	ABA	[96]

**Table 2:** Mutations of strigolactone (SL) and karrikin (KAR)-related genes that alter the effects in various plants species under different abiotic stresses.

Genotypes	Types of Stresses	Mutants or Transgenes	Effects	Interactions with Phytohormones	References
Arabidopsis	Drought stress and salinity	*max2-3, max2-4, max3-11, max3-12, max4-7,* and *max4-8*	SLs positively regulate plant responses to drought and salt stress	ABA and CK	[41]
Arabidopsis	Drought stress	*max2-1, max2-2, max1, max3,* and *max4*	*MAX2* play an important role in plant responses to abiotic stress	ABA	[48]
Lotus japonicus	Phosphate starvation and osmotic stress	*LjCCD7-*silenced line *(Ljccd7)*	SLs contribute to drought resistance in *Lotus japonicus*	ABA	[143]
Arabidopsis	Wounding, heat, UV-B, salinity	Wild type	Abiotic stresses responses	ABA, CK, IAA, BR, ET, GA, and MeJA	[205]
Tomato	Drought	*SlCCD7-*silenced line *(Ljccd7)*	Low levels of SLs in roots act as components of the systemic signal of drought stress	ABA	[38]
Tomato	Drought and AMF	Wild type	AMF induces SL biosynthesis under drought and improves drought tolerance	ABA	[46]
*Festuca arundinacea*	PEG-induced drought stress	Wild type	Drought-inhibition of tiller development and growth in grass species are associated with SL accumulation and signaling		[206]
Rapeseed	salinity	Wild type	Salinity depresses the shoots and roots growth, whereas GR24 improves the growth under salt stress		[42]
*Sesbania cannabina*	Salinity and AMF	Wild type	SLs enhance salt stress tolerance, and the H_2_O_2_-induced SL accumulation was accompanied by increased salt tolerance		[207]
*Rice*	Drought stress	*d10, d17, d27,* and *d3, D27-*overexpressing plants	SL biosynthesis/perception interferes with ABA formation, and D27 plays a crucial role in determining ABA and SL content	ABA	[40]
*Sesbania cannabina*	Salinity and AMF	Wild type	ABA is regulating the induction of salt tolerance by SL in AM seedlings	ABA	[39]
Arabidopsis	Dark	*max1-1, max3-9, max4-11, Atd14-1,* and *max2-4*	ET synthesis and consequent SL synthesis are required for the efficient progression of dark-induced leaf senescence.	ET	[34]
Arabidopsis	Phosphate deficiency	*max2-1* and *max4-1*	SLs regulate the response of plants to low Pi	Auxin	[34]
Rice	Phosphate- and nitrate-deficiency	*d3, d10,* and *d27*	SLs affect root growth in rice under phosphate and nitrate limitation by decreasing auxin transport from shoots to roots	Auxin	[157]
Rice	Phosphate- and nitrate-deficiency	*d3, d10,* and *d53*	SLs involve in NO-activated elongation of seminal root under nitrogen and phosphate deficiency conditions.		[193]
Arabidopsis and pea	Dark chilling	*max2-1, max3-9,* and *max4-1* in *Arabidopsis; rms5-3, rms4-1,* and *rms3-1* in *pea*	SLs positively regulate chilling tolerance in pea and in Arabidopsis		[44]
Arabidopsis	Drought stress	*kai2-2, kai2-4,* and *d14-2*	The KAR receptor KAI2 promotes drought resistance	ABA	[8]
Arabidopsis	Osmotic stress and salinity	*kai2-2, d14-1, max2-1* and *max2-7*	Karrikin-KAI2 signaling system can protect against abiotic stress	GA and ABA	[63]

## Abbreviations

SL	Strigolactone
KAR	Karrikin
ABA	Abscisic acid
CK	Cytokinin
GA	Gibberellic acid
ET	Ethylene
SA	Salicylic acid
JA	Jasmonate
BR	Brassinosteroid
D10	DWARF10
D14	DWARF14
D27	DWARF27
D53	DWARF53
KAI2	KARRIKIN INSENSITIVE 2
MAX2	MORE AXILLARY GROWTH 2
SMAX1	SUPPRESSOR OF MAX2 1
SMXL	SMAX1-LIKE
LBO	LATERAL BRANCHING OXIDOREDUCTASE
CCD	CAROTENOID CLEAVAGE DIOXYGENASE
RMS	Ramosus
N	Nitrogen
P	Phosphate
CKX	CYTOKININ OXIDASE
CL	Carlactone
AMF	Arbuscular mycorrhizal fungus
TMB	Trimethylbutenolide
SW	Smoke-water
